# Prevalence of self-reported tuberculosis, knowledge about tuberculosis transmission and its determinants among adults in India: results from a nation-wide cross-sectional household survey

**DOI:** 10.1186/1471-2334-13-16

**Published:** 2013-01-17

**Authors:** Chandrashekhar T Sreeramareddy, H N Harsha Kumar, John T Arokiasamy

**Affiliations:** 1 Department of Clinical Sciences, Faculty of Medicine and Health Sciences, Universiti Tunku Abdul Rahman, Sungai Long, Malaysia; 2 Department of Community Medicine, Kasturba Medical College, Mangalore, India; 3 Department of Community Medicine, International Medical University, Kuala Lumpur, Malaysia

## Abstract

**Background:**

Knowledge about symptoms and transmission of tuberculosis determines health seeking behavior and helps in prevention of tuberculosis transmission in the community. Such data is useful for policy makers to formulate information, education and communication strategies for tuberculosis control.

**Methods:**

A secondary data analysis of India demographic and health survey, 2005/6 was carried out. Questions about self-reported tuberculosis, transmission and curability of tuberculosis were analysed. Correct knowledge (without misconceptions) about tuberculosis transmission was used as a dependant variable and the explanatory variables tested were: demographic data, education, wealth quintiles, frequency of exposure to media and the curability of tuberculosis. Determinants of correct knowledge without misconceptions were tested by univariate and multivariate analyses using national weighting factor to adjust for complex sampling design.

**Results:**

A total of 109,070 households (response rate of 93.5%) and 198,718 participants (response rate of 91.6%) completed the survey. The samples of men and women interviewed were 74,360 and 124,358 respectively. Prevalence rate of self-reported tuberculosis was 445 per 100,000 usual household residents and 4.60 per 1,000 participants. The number of respondents who had “*heard of an illness called tuberculosis*” was 177,423 (89.3%). Of these 47,487 (26.8%) participants did not know and 55.5% knew about the correct mode of tuberculosis transmission i.e. “*Through the air when coughing or sneezing*”. The common misconceptions about transmission were “Through food” (32.4%), “Sharing utensils” (18.2%), and “Touching a person with tuberculosis” (12.3%). Only 52,617 (29.7%) participants had correct knowledge without misconceptions. Being male (aOR 1.17, 95% CIs 1.14, 1.21), being a Hindu (aOR 1.20, 95% CIs 1.14, 1.26) or Muslim (aOR 1.26, 95% CIs 1.18, 1.34), listening to radio (aOR 1.08, 95% CIs 1.04, 1.13) and “Tuberculosis can be cured” (aOR 1.47, 95% CIs 1.41, 1.53) were associated with correct knowledge without misconceptions.

**Conclusions:**

Knowledge about tuberculosis transmission is very poor and misconceptions still exist. Among the traditional mass media, the frequency of listening to radio was associated with correct knowledge about tuberculosis transmission. Strategies to deliver information, education and communication campaigns could be improved.

## Background

Tuberculosis (TB) is a global public health problem with a third of the world's population infected with *Mycobacterium tuberculosis*[1]. TB accounted for 9.4 million new cases, 11.1 million prevalent cases and 1.3 million deaths during the year 2008 [2]. In low- and middle-income countries (LMICs), TB stands third among the leading causes of adult mortality after human immunodeficiency virus and ischaemic heart disease [3]. Out of the 22 high-burden countries for TB, eleven are situated in Asia, while nine are in Africa. These countries account for approximately 80% of the total cases worldwide. In Asia, India is also a high-burden country along with China [2]. The average prevalence of all forms of tuberculosis and smear-positive cases was estimated at 5.05, and 2.27 per 1,000 population respectively with an annual incidence of smear-positive cases at 84 per 100,000 population [4]. In India during 2010, the estimated prevalence rate for all forms of TB was 283 per 100,000 population. In India, each year nearly 2.2 million people develop TB disease, of which one million are new smear-positive cases and half a million people die from TB [5].

Up until now, early case detection and treatment of cases is the only and most effective method of TB control [6]. The current TB control strategy with Directly Observed Treatment; Short course (DOTS) is reported to be successful in terms of treatment success rate [7]. However, passive case detection strategy followed under DOTS may not have achieved the case detection rate of at least 70% [8,9]. In India, a passive case detection method is followed under the Revised National Tuberculosis Control Program (RNTCP) for TB case-finding. This may be the reason for the delayed presentation of TB patients at healthcare facilities where diagnostic facilities for TB are available [10]. Two systematic reviews on the delay in diagnosis of TB have highlighted that longer patient delays may be due to lack of awareness about TB symptoms among the population [11,12]. Therefore, TB control programs have recognized the importance of providing information, education and communication (IEC) to improve the knowledge about TB and to influence change in health-care seeking behavior among both TB patients and the general public. In India, though an integral component of RNTCP, IEC activities were minimal and scattered up until 2001. Since 2001, a sustained intensified IEC campaign is being done [7]. Recently, a STOP-TB initiative has adopted the strategy of advocacy, communication and social mobilization to support country TB control programmes [7,13,14].

Knowledge about TB symptoms, modes of transmission of TB, and misconceptions about TB transmission among the general public may have an impact on health-care seeking behavior [15,16]. A sound understanding of the knowledge about symptoms and misconceptions about TB transmission in the general population is important to formulate messages for health education. Surveys about knowledge, attitude and practice may yield data for impact assessment of the ongoing IEC campaign [16-19]. A few studies have reported about the knowledge of TB transmission in the general population, medical interns and physicians [15,20-23]. A study about knowledge of TB transmission among ever-married women in Bangladesh has been reported [24]. However, there are no reports on a nationally representative sample from India. We analyzed such information available in the data from India Demographic Health Survey 2005/6. Our objectives were to provide prevalence estimates of self-reported TB, to assess the knowledge and misconceptions about TB transmission and to identify the determinants of ‘*correct knowledge’* about TB transmission.

## Methods

### Data source

India Demographic and Health Survey, 2005/6 (IDHS 2005/6) which is also called as the National Family and Health Survey-III (NFHS-III) was conducted between November 2005 and August 2006 under the scientific and administrative supervision of the International Institute for Population Sciences (IPPS), Mumbai and ORC (Opinion Research Corp.) Macro International. Trained interviewers collected data on demographic factors, socio-economic factors and health status from a nationally representative probability sample of households. Data were collected according to a standard protocol of Demographic and Health Survey (DHS) which had three core survey questionnaires i.e. the Household Questionnaire, the Woman’s Questionnaire and the Man’s Questionnaire. The questionnaires were translated into 18 local languages; field tested and were subsequently back-translated to English. These questionnaires were used in all 29 states of India. In each of these states, the questionnaires used were bilingual i.e. questions were in the principal language of the state, and in English. To minimize language barriers, the survey was administered by trained interviewers either in English or in the principal language of the state or the preferred language of the household.

### Sampling methods

**Figure 1:**
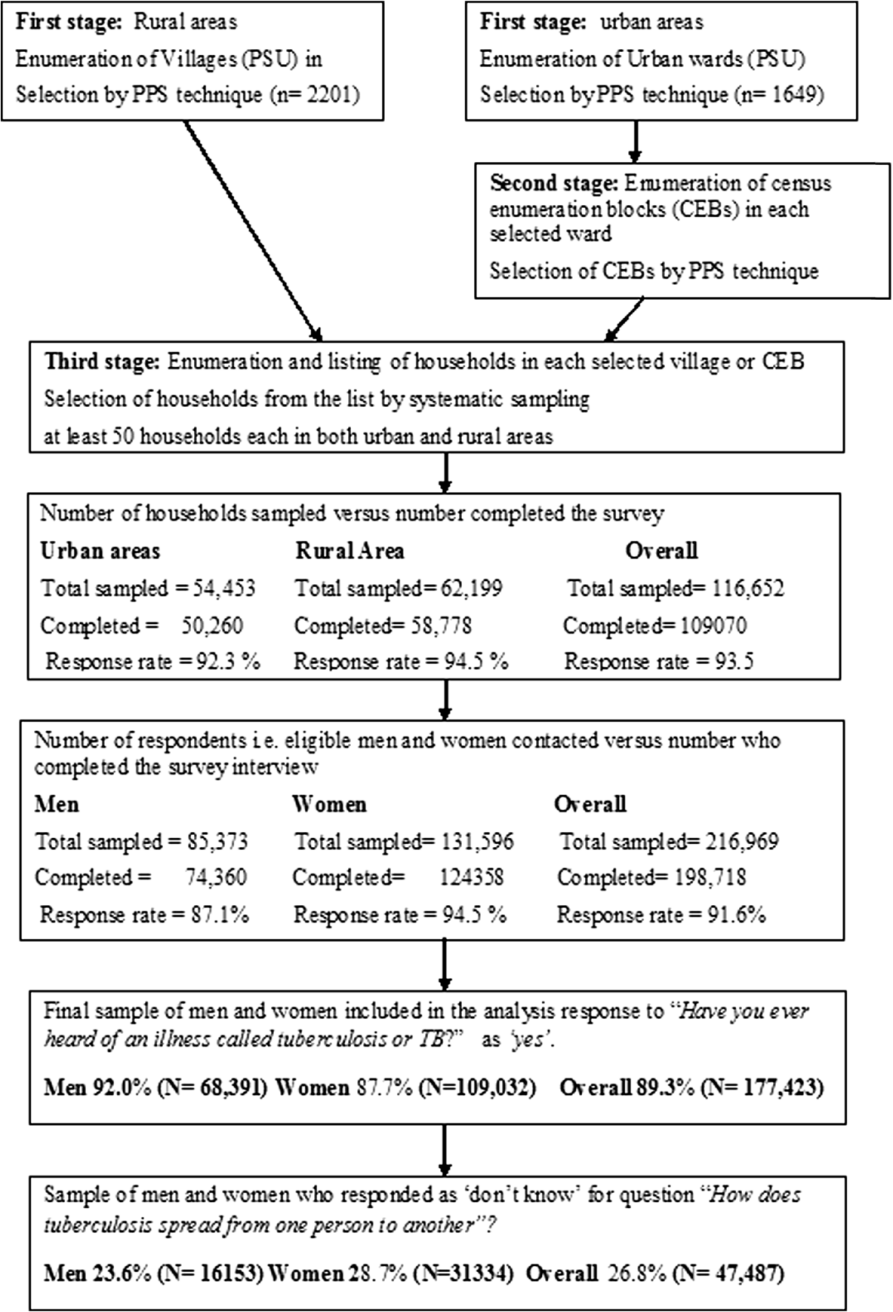
Sampling method and selection of sample for analysis.

A nationally representative sample of households was selected through a stratified, multistage cluster sampling method. By this method, the households were selected by the two-stage probability proportional to size (PPS) method in rural areas and the three stage PPS sampling method in urban areas (Figure 1). A uniform sampling design was used across all the 29 states. Urban and rural samples were drawn separately and were proportionate to the population size of the state, unless oversampling was required for an area or a group. For both urban and rural areas, geographic sampling units obtained were villages in rural areas and census blocks/wards in urban areas. A random household sampling method with household as primary sampling unit (PSU) was undertaken in chosen geographic sampling units. Further details of sampling design and sample size obtained for this analysis are given in Figure 1. Details about training of the survey team, survey management and quality control measures are separately documented in the country reports published by ORC Macro International [25].

### Ethical issues and consent

Independent review boards (IRBs) of IPPS and ORC Macro International reviewed the DHS standard protocols, data collection tools, procedures, and provided the ethical approval. Trained interviewers informed the survey participants that participation in the survey was voluntary. They were also assured about confidentiality of the information to be provided and could opt not to answer any of the questions during the interview. Informed consent was obtained from each survey participant.

### Variables

In the Household Questionnaire section, the main respondent of the household was asked to list all the usual residents living the house. The main respondent was asked *“Do any usual resident of your household suffer from tuberculosis?”* If the response was *‘yes’,* they were then asked *“Who suffers from tuberculosis?”* Responses to these questions were used to estimate the prevalence of self-reported TB. Both men and women were asked two main questions about TB in their respective questionnaires. The first one was *“Have you ever heard of an illness called tuberculosis or TB*?” If the response to the first question was *‘yes’,* then a second question *“How does tuberculosis spread from one person to another?”* was asked to assess their knowledge about TB transmission. For the second question, the respondents were given following options. The responses were marked as *‘yes’* or *‘no’*.

a) Through the air when coughing or sneezing

b) Through spit/sputum/stepping on spit

c) Through sharing utensils

d) Through sharing clothes/bed sheets/towel

e) Through smoking bidis/cigarette/tobacco

f) Through touching a person with tuberculosis

g) Through food

h) Through sexual contact

i) Through mosquito bites

j) Don’t know

For our analysis, the response to option a) “*Through air when coughing or sneezing”* was considered as correct knowledge about TB transmission. The options from ‘b’ to ‘i’ were considered as misconceptions. We created a new variable by re-coding responses to question about TB transmission. This provided us a sample of individuals who responded as ‘*yes*’ to the option “*Through air when coughing or sneezing”* and responded as ‘*no*’ to other options about modes transmission for TB. The recoding gave us a new variable *“correct knowledge without misconception”.* The other questions asked about TB were *“Can tuberculosis be cured?”* and *“If a member of your family got tuberculosis, would you want it to remain a secret or not?”*

### Dependant variable

Based on the responses to questions explained above regarding TB transmission, a binary dependant variable ‘correct knowledge about TB transmission’ was created for multivariable analysis. ‘Correct knowledge about TB transmission’ was defined as follows: If a participant had correct knowledge (i.e. TB transmission *“Through the air when coughing or sneezing”*) but had no misconceptions about TB transmission. Incorrect knowledge was considered either if the respondent had misconceptions despite responding as ‘*yes*’ for TB transmission *“Through the air when coughing or sneezing”*, or had only misconceptions or responded as "*Don’t know"*.

### Explanatory variables

We tested the association between *correct knowledge* about TB transmission and the following explanatory variables: age, gender, highest level of education attained, marital status, religion, type of residence (urban/rural), wealth index, national region of residence and exposure to mass media (frequency of reading newspaper/magazine, listening to radio, and watching television). Wealth index is a relative index of household wealth. It was calculated based on a standard set of household assets, dwelling characteristics and ownership of consumer items according to observation by the interviewer. The individuals were ranked on the basis of their household score and divided into quintiles. The first quintile is the poorest 20% of the households and fifth quintile is the wealthiest 20% of the households. In India DHS 2006, the respondents were asked about the frequency of reading newspaper/magazine, listening to radio, and watching television. The response options were ‘not at all’, ‘less than once a week’, ‘at least once a week and ‘almost every day’. We also tested *“Can tuberculosis be cured?”* for association with correct knowledge about TB transmission. For national region of residence, the 29 states were grouped as East, West, Central, North, South and North-East. National regions of residence were categorized based on those created by the International Institute for Population Sciences. These categories are as follows: North (New Delhi, Haryana, Himachal Pradesh, Jammu/Kashmir, Punjab, Rajasthan, Uttaranchal), Central (Madhya Pradesh, Uttar Pradesh, Chhattisgarh), East (Bihar, Orissa, West Bengal, Jharkhand), North-East (Arunachal Pradesh, Assam, Manipur, Meghalaya, Mizoram, Nagaland, Sikkim, Tripura), West (Goa, Gujarat, Maharashtra), South (Andhra Pradesh, Karnataka, Kerala, Tamil Nadu).

### Data analysis

Response rates for households contacted and individuals invited to participate in the survey were calculated. Prevalence rates of tuberculosis per 100,000 usual residents in the household and per 1,000 respondents were calculated. For comparison of prevalence rates among the respondents by selected demographic variables, Chi-square test was used to test the statistical significance. The percentage of participants responding as *‘yes’* for questions about TB and its modes of transmission were calculated. The dichotomous variable *‘correct knowledge about TB transmission*’ was used as a dependant variable for multivariable analysis to identify the determinants of *‘correct knowledge about TB transmission’*. The explanatory variables used were demographic factors (age, gender, religion and marital status), socio-economic factors (educational attainment and wealth quintiles), spatial factors (urban/rural residence, region/state) and other factors (*“Can TB can be cured?”),* frequency of reading newspaper, watching television and listening to radio). National weighting factor was used to adjust for multistage sampling used in DHS. Complex samples analysis option was used in statistical package for Social Sciences (SPSS) version 17. Univariate analyses followed by multivariable analyses were carried out. In multivariate analyses adjusted odd ratios (aOR) and their 95% confidence intervals (95% CIs) were calculated. For all these analyses a p-value <0.05 was considered as significant.

## Results

### Household and participants response rates

Of the 116,652 households that were sampled, 109,070 completed the survey giving an overall household response rate of 93.5%. The response rates in urban and rural areas were 92.3% and 94.5% respectively. A total 216,969 individuals were contacted of which 198,718 responded giving an overall response rate of 91.6%. The participant response rate of 94.5% for women was higher than 87.1% for men (Figure 1).

### Prevalence of self-reported tuberculosis

The overall prevalence rate for tuberculosis was 445 per 100,000 usual household residents. The prevalence rate in rural areas (502 per 100,000 usual household residents) was significantly higher than the urban areas (319 per 100,000 usual household residents). Among the total of 198,754 participants, 915 reported to be suffering from tuberculosis giving an overall prevalence rate of 4.60 per 1,000 participants. The prevalence of self-reported TB according to important explanatory variables is shown in Table 1. The prevalence was higher among those aged 40 years and above, men, poorer and poorest (by wealth quintiles), and those who did not have any education. Among the regions of India, North-Eastern and Eastern regions had higher prevalence than the rest of India. All these comparisons were statistically significant (Table-1).

**Table 1:** Prevalence of self-reported Tuberculosis among adult men (aged 15–59 years) and women (aged 15–49 years) by selected demographic and socio-economic characteristics

	**Sample (N)**	**Number reporting TB**	**Prevalence per 1000 population**	**95% CIs**
**Age**				
≤25 years	72300	187	2.58	2.22, 2.96
26-40 years	84639	423	4.99	4.52, 5.47
>40 years	41815	305	7.29	6.48, 8.11
**Gender**				
Male	72369	443	6.12	5.55, 6.69
Female	124385	472	3.79	3.45, 4.14
**Type of residence**				
Urban	95160	353	3.71	3.32, 4.10
Rural	103594	562	5.43	4.98, 5.87
**Education**				
No education	50465	411	8.14	7.36, 8.93
Primary	29230	160	5.47	4.63, 6.32
Secondary	94627	308	3.25	2.89, 3.62
Higher	24389	36	1.48	0.99, 1.96
**Wealth Quintiles**				
Poorest	21162	119	5.62	4.62, 6.63
Poorer	27930	192	6.87	5.91, 78.4
Middle	38547	220	5.71	4.96, 6.46
Rich	49482	185	3.74	3.20, 4.28
Richest	61633	119	1.93	1.58, 2.28
**Region of India**				
Northern India	31584	89	2.82	2.23, 3.40
North-eastern India	34776	233	6.70	5.84, 7.56
Central India	37987	199	5.24	4.51, 5.96
Western India	27707	126	4.55	3.76, 5.34
Eastern India	24606	146	5.93	4.97, 6.89
South India	42094	122	2.89	2.38, 3.41

*all the comparison were statistically significant p < 0.01.

### Knowledge about TB transmission

Overall, a high proportion (89.3% i.e.177, 423) of the respondents had *“heard of an illness called Tuberculosis (TB)”*. The proportion of men (92% i.e. 68,391) who had *“heard of an illness called Tuberculosis (TB)”* was higher than that of women (87.7% i.e. 109,032). Among those who had heard about TB, 26.8% (47,487) responded as "*Don’t know"* for the question about transmission of TB (men 23.6% versus women 28.7%). Only 55.5% of them knew about the correct mode for transmission of TB i.e. *‘Through the air when coughing or sneezing’*. Various misconceptions about TB transmission prevailed among the participants. Among these, TB transmission through food (32.4%), sharing utensils (18.2%), touching a person with TB (12.3%) were very common. Other misconceptions according to sex are shown in Table 2. By our operational definition only 29.7% (i.e. 52, 617) of the participants had *correct knowledge about TB transmission*. The majority (83.5%) of participants knew that TB can be cured but 15.6% of participants said that they “*Would keep it a secret from neighbors if a member of their family got tuberculosis”*.

**Table 2:** Percentage of adult men and women in India responding as ‘yes’ to questions about tuberculosis transmission

		**Men (N = 68,391)**	**Women(N = 109,032)**	**Overall (N = 177,423)**
1	How does tuberculosis spread from one person to another?			
	a)Through the air when coughing or sneezing	56.3	55.0	55.5
	b)Through food	28.7	34.9	32.4
	c)Through sharing utensils	16.0	19.7	18.2
	d)Through touching a person with TB	11.8	12.7	12.3
	e)Through sexual contact	5.9	5.0	5.3
	f)Through mosquito bites	1.9	1.4	1.6
	g)Blood/blood transfusions	1.1	0.7	0.9
	h)Through smoking bidis/cigarette/tobacco	1.1	0.5	0.7
	i)Through sharing clothes/bed sheets/towel	0.6	0.6	0.6
	j)Through spit/sputum/stepping on spit	0.4	0.7	0.5
	k)Others	1.7	1.7	1.7
	l)Don’t know	23.6	28.7	26.8
2	Can tuberculosis be cured?	86.5	81.5	83.5
3	If a member of your family got tuberculosis, would you want it to remain a secret from the neighbors or not?	15.8	15.4	15.6

### Determinants of correct knowledge of TB

Determinants of correct knowledge about TB transmission among participants were assessed by univariate and multivariate analyses (Table 3). By univariate analysis, male gender, being single, living in urban areas, following Hindu or Muslim religion, have higher education and being richer or richest by wealth quintiles were associated with ‘*correct knowledge about TB transmission*’. In addition, reading newspaper every day, watching television everyday and knowing that TB can be cured were also associated with correct knowledge. After adjustment for interactions among the explanatory variables being male (aOR 1.17, 95% CIs 1.14, 1.21), being a Hindu (aOR 1.20 95% CIs 1.14, 1.26) or Muslim (aOR 1.26, 95% CIs 1.18, 1.34) and ‘*TB can be cured*’ (aOR 1.47, 95% CIs 1.41, 1.53) were associated with correct knowledge while education, and wealth quintiles though remained significant, the effect size was very small. Frequency of reading news paper/magazines and watching television were not significant; frequency of listening to radio (aOR 1.08, 95% CIs 1.04, 1.13) was associated with correct knowledge. Age of the respondent was not associated with correct knowledge by both univariate and multivariate analysis. Participants from Eastern, North-Eastern and Central India were less likely to have correct knowledge about TB transmission while those from Western India were more likely to have correct knowledge about TB transmission (Table 3).

**Table 3:** Determinants of correct knowledge about Tuberculosis transmission among adult men (aged 15–59 years) and women (aged 15–49 years) by univariate and multivariable analyses

	**Unadjusted odds ratio (95% CIs)**	**Adjusted odds ratio (95% CIs)**
**Age**		
>40 years	1	1
26-40 years	1.02 (0.98, 1.06)	0.99 (0.96, 1.03)
≤25 years	1.01 (0.97, 1.05)	0.98 (0.93, 1.03)
**Gender**		
Female	1	1
Male	1.23 (1.20, 1.27)	1.17 (1.14, 1.21)
**Current marital status**		
Divorced/separated/widowed	1	1
Single	0.84 (0.77, 0.91)	0.94 (0.86, 1.04)
Married	0.97 (0.80, 1.06)	1.01 (0.93, 1.10)
**Religion**		
Others	1	1
Muslim	1.52 (1.43, 1.61)	1.26 (1.18, 1.34)
Hindu	1.37 (1.30, 1.44)	1.20 (1.14, 1.26)
**Type of residence**		
Urban	1	1
Rural	0.80 (0.78, 0.83)	0.98 (0.95, 1.02)
**Educational attainment**		
No education	1	1
Primary	1.17 (1.13, 1.23)	1.07 (1.02, 1.12)
Secondary	1.42 (1.34, 1.50)	1.10 (1.03,1.18)
Higher	1.62 (1.55, 1.71)	1.08 (1.004, 1.16)
**Wealth quintiles**		
Poorest	1	1
Poorer	1.11 (1.07, 1.53)	1.02 (0.98, 1.06)
Middle	1.29 (1.24, 1.35)	1.09 (1.03, 1.14)
Richer	1.51 (1.44, 1.58)	1.12 (1.05, 1.19)
Richest	1.59 (1.51, 1.67)	1.12 (0.99, 1.14)
**Region of India**		
Northern India	1	1
North-eastern India	0.58 (0.55, 0.61)	0.58 (0.54, 0.61)
Central India	0.79 (0.75, 0.85)	0.85 (0.80, 0.90)
Western India	1.27 (1.19, 1.35)	1.21 (1.14, 1.28)
Eastern India	0.52 (0.49, 0.55)	0.54 (0.51, 0.58)
South India	1.02 (0.96, 1.08)	1.01 (0.95, 1.08)
**Watches television**		
Not at all	1	1
Once or less in a week	1.30 (1.26, 1.35)	0.99 (0.96, 1.03)
Everyday	1.45 (1.39, 1.50)	0.977 (0.93, 1.03)
**Reads newspapers/magazines**		
Not at all	1	1
Once or less in a week	1.27 (1.31)	1.05 (1.003, 1.09)
Everyday	1.55 (1.50, 1.61)	1.05 (0.99, 1.11)
**Listens to the radio**		
Not at all	1	1
Once or less in a week	0.94 (0.91, 0.98)	1.043 (1.01, 1.08)
Everyday	1.03 (0.99, 1.06)	1.084 (1.04, 1.13)
**Can TB be cured?**		
No/don’t know	1	1
Yes	1.44 (1.39, 1.50)	1.47 (1.41, 1.53)

## Discussion

Our analyses revealed that the majority of the participants had heard about TB, though their knowledge about TB transmission was low. It also provided national level prevalence estimates for TB, which is comparable to routine programmatic data. The highlights of our analyses were that nearly half the population knew that TB transmission occurs through air when coughing or sneezing but only a quarter knew about correct mode of TB transmission i.e. without having any misconceptions about it. Encouragingly, the majority of participants knew that TB can be cured and this was associated with having correct knowledge about TB transmission. Among the traditional mass media, only listening to the radio was associated with correct knowledge about TB transmission. From our results, we may interpret that widespread publicity about DOTS may have improved the general awareness about TB, and specifically about the cure for TB. Our results may be useful for managers and policy makers of RNTCP and emphasized the need for evaluation of the IEC activities undertaken by RNTCP.

The strength of our analyses was the calculation of national level prevalence estimates for self-reported TB, and knowledge about transmission of TB from a large representative sample of adult men and women. Our report also includes misconceptions and the stigma about TB which are scarce in the literature. Despite these strengths, we had some limitations and our results should be interpreted with caution. The DHS questionnaire inquired mainly about the modes of transmission but not about TB symptoms and treatment availability. Respondents were not asked about the sources of information about TB. Estimates of self-reported TB in rural areas may have been flawed due to lack of awareness about TB symptoms, low education and the stigma attached with the disclosure of TB. DHS interviewers were well trained to extract such information. However, there may have been an over-estimation of self-reported TB and DHS did not have any means to verify self-reported TB by laboratory tests. Assessing the determinants of correct knowledge about TB transmission using a cross-sectional data lacks temporality to interpret a cause-effect relationship.

The prevalence of self-reported TB in our analysis was more than the estimated TB prevalence reported by WHO and from the annual reports of the Ministry of Health (MOH), Government of India (GOI) [5,9]. It is not clear from our analysis, if there was under-reporting because the questions asked during the survey were not specific to pulmonary and extra-pulmonary TB. Moreover, extra-pulmonary TB which is less known in the general population may not have been reported by the survey respondents. Despite the skepticism regarding self-reports about health and disease status in surveys, we believe that self-reported TB in DHS is reliable [26,27]. The data are comparable to existing reports on TB burden in India [5,9]. The socio-economic patterning of TB was in accordance with the previous reports [28-30]. TB experts have recommended that socio-economic data should be measured during TB surveys [31]. However we did not perform further analysis on determinants of TB using other explanatory variables since in this survey TB was a self-report. Higher prevalence of TB in rural areas and in North-Eastern and Eastern states is also in accordance with annual reports of MOH, GOI [9].

Several studies from different countries about awareness, perceptions, attitudes, and treatment seeking behaviors for TB have reported that awareness about TB in the general population is poor and treatment seeking behavior is not appropriate [15,21,23,32-36]. However, most of these except our study and a report from Bangladesh [24] were small scale surveys and lacked the implications on policy making at the national level. A good knowledge about TB symptoms in the general population may help to improve health-care seeking behavior of patients [10]. Knowledge about transmission of TB is also important to protect oneself from infection with TB by following cough etiquette and respiratory hygiene which are critical in preventing TB transmission [37]. Our results suggest that prevention of TB transmission in the general population of India is less likely since people did not know the correcmode of TB transmission and people had misconceptions about it. In the neighboring country of Bangladesh, the proportion of women knowing correctly about TB transmission was much lower [24]. Correct knowledge about TB transmission was associated with education and income, but among mass media, only the frequency of listening to radio was associated with correct knowledge which was similar to the results reported from Bangladesh [24]. Inadequate knowledge about TB transmission in India may be due to lack of IEC messages about TB transmission before and during the time of DHS survey i.e. 2005–06. The IEC messages which focused on symptoms, curability and availability of treatment (DOTS) were disseminated by the RNTCP, which implements TB control activities in India. The IEC activities were meant to achieve the targets in terms of case detection rate and cure rates set by RNTCP. In pursuit of these targets, RNTCP program planners may have missed the inclusion of messages about TB transmission. However, recently, the pamphlets about facts of TB contain the message *“TB is spread through coughing or sneezing of a TB patient.”* While case detection and cure of TB is secondary prevention, prevention of TB transmission before infectious cases are diagnosed and treated is primary prevention. We emphasize that primary prevention cannot be overlooked considering the reports about diagnostic delays in high burden countries [11,12].

### Policy implications

Currently, knowledge about symptoms and transmission of TB, stigma about TB, and health seeking behavior for TB are not measured in program evaluation. Inclusion of such indicators into periodic program evaluation may serve as a guide to improve the program’s performance. Two studies about the evaluation of IEC activities of RNTCP done in New Delhi have reported about issues other than TB transmission and have called for an improvement in IEC strategies [16,18]. Lack of association of correct knowledge about TB transmission with traditional mass media such as television, newspapers and magazines shows the need for using other means to disseminate IEC messages about TB [16,18]. This may be due to lower levels of literacy prevailing in India. People belonging to the poorest wealth quintiles were more likely to have TB while the frequency of listening to radio was associated with knowledge about TB transmission. Since radio is more affordable to the poorest people, radio may be used to intensify IEC activities. TB control program planners should also consider primary prevention in addition to early diagnosis and treatment. The regional differences in TB prevalence and knowledge of TB transmission should be considered in program planning. Areas with high TB prevalence and lower knowledge may need advocacy, communication and social mobilization as recommended by STOP-TB initiative [13,14,19]. Nation-wide surveys like DHS provide a good opportunity to study further about TB knowledge and health behavior towards TB. Future DHS surveys may include questions about the knowledge of TB symptoms and treatment seeking behavior among people having cough lasting three weeks or more. As DHS surveys are not done frequently, RNTCP could consider inclusion of TB knowledge, and health-care seeking behavior for TB in their program evaluation to assess the impact of IEC activities on the diagnosis of TB [17].

## Conclusion

Knowledge about TB transmission in the general population of India is very poor and misconceptions about TB transmission prevailed. Among traditional mass media, only the frequency of listening to radio was associated with knowledge about TB transmission. TB control program should include more information about TB transmission in its IEC messages and alternative mass media such as radio could be considered when delivering messages about TB.

## Competing interests

The authors declare that they have no competing interests.

## Authors’ contributions

CTS: Conceptualized the research, conducted the data analysis, interpreted the results and wrote the first draft of the manuscript for publication; HNHK: Helped conceptualizing the research, planned data analysis and revised earlier drafts of the manuscript; JTA: Assisted in drafting the manuscript, commented draft versions of the manuscript for publication. All the authors read and approved the final version of the manuscript to be submitted for publication in a scientific journal.

## Pre-publication history

The pre-publication history for this paper can be accessed here:

http://www.biomedcentral.com/1471-2334/13/16/prepub
